# Acute exacerbation of immunoglobulin A nephropathy complicated by alveolar hemorrhage after coronavirus disease 2019 vaccination: A case report

**DOI:** 10.1097/MD.0000000000036091

**Published:** 2023-11-17

**Authors:** Takahiro Uchida, Takashi Sakai, Takahiko Hoshino, Aki Kojima, Osamu Konno, Muneharu Yamada, Hitoshi Iwamoto, Takashi Oda

**Affiliations:** a Department of Nephrology and Blood Purification, Kidney Disease Center, Tokyo Medical University Hachioji Medical Center, Hachioji, Tokyo, Japan; b Department of Kidney Transplantation Surgery, Kidney Disease Center, Tokyo Medical University Hachioji Medical Center, Hachioji, Tokyo, Japan.

**Keywords:** alveolar hemorrhage, COVID-19, COVID-19 vaccination, IgA nephropathy, renal transplantation

## Abstract

**Rationale:**

Reports have suggested a relationship between coronavirus disease 2019 (COVID-19) vaccination and new-onset or recurring renal diseases, of which immunoglobulin A (IgA) nephropathy is a representative disease. Alveolar hemorrhage in patients with IgA nephropathy is rare but reportedly has a high mortality and morbidity. To our knowledge, there have been no reports regarding the development of IgA nephropathy with alveolar hemorrhage following COVID-19 vaccination.

**Patient’s concern:**

A 23-year-old Japanese man presented with hemoptysis and peripheral edema a few days after receiving a second dose of a COVID-19 mRNA vaccine. Severe renal failure and alveolar hemorrhage were noted thereafter, and renal biopsy showed crescentic glomerulonephritis with mesangial proliferation accompanied by mesangial electron-dense deposits containing IgA. Renal biopsy tissue also showed chronic histological changes suggestive of acute exacerbation of preexisting IgA nephropathy.

**Diagnosis:**

The diagnosis of IgA nephropathy complicated by alveolar hemorrhage was made.

**Interventions and outcomes:**

Renal function did not recover despite treatment with high-dose steroids; the patient was maintained on hemodialysis and eventually underwent successful renal transplantation.

**Lessons:**

The present case suggested that although extremely rare, severe renal failure requiring renal replacement therapy could occur in patients with IgA nephropathy after COVID-19 vaccination. Future accumulation of similar cases is needed to predict the risk of renal injury following vaccination.

## 1. Introduction

The introduction of vaccinations against coronavirus disease 2019 (COVID-19), caused by severe acute respiratory syndrome coronavirus 2 (SARS-CoV-2) infection, greatly reduced the burden of the global COVID-19 pandemic. A substantial decrease in COVID-19-related mortality was reported over serial waves of the pandemic, attributed to changes in the virus variants and the widespread use of COVID-19 vaccines.^[1]^

On the other hand, COVID-19-related mortality and morbidity remain high in specific subgroups, including patients with renal diseases.^[2]^ Adequate vaccination is therefore required for such patients, but the development or relapse of renal disease following COVID-19 vaccination is a concern. Several renal diseases have been reported, with minimal change disease (MCD) and immunoglobulin (Ig)A nephropathy as representative disease.^[3]^ Most reported cases followed a mild or self-limiting clinical course and the benefits of COVID-19 vaccination outweigh the risks^[3–5]^; however, both precise pathogenesis and appropriate approaches for postvaccination renal diseases remain unclear.

Among the several extrarenal symptoms IgA nephropathy presents with, alveolar hemorrhage is an extremely rare manifestation that has high mortality and morbidity.^[6]^ To our knowledge, no cases of IgA nephropathy accompanied by alveolar hemorrhage after COVID-19 vaccination have been reported.

Herein, we report a case of IgA nephropathy complicated by alveolar hemorrhage after administration of the second dose of a COVID-19 mRNA vaccine. The disease course in this patient was atypical, in that the patient eventually required maintenance dialysis despite prompt treatment with high-dose steroids.

## 2. Case presentation

A 23-year-old Japanese man who received a second dose of a COVID-19 vaccine (mRNA-1273) a few days ago presented to a nearby clinic with hemoptysis and peripheral edema. Anemia was noted, and chest X-ray revealed patchy opacities. The patient was then referred to our hospital. The patient had undergone a medical checkup 5 years earlier and had no significant medical history or family history of renal disease.

**Figure 1.**
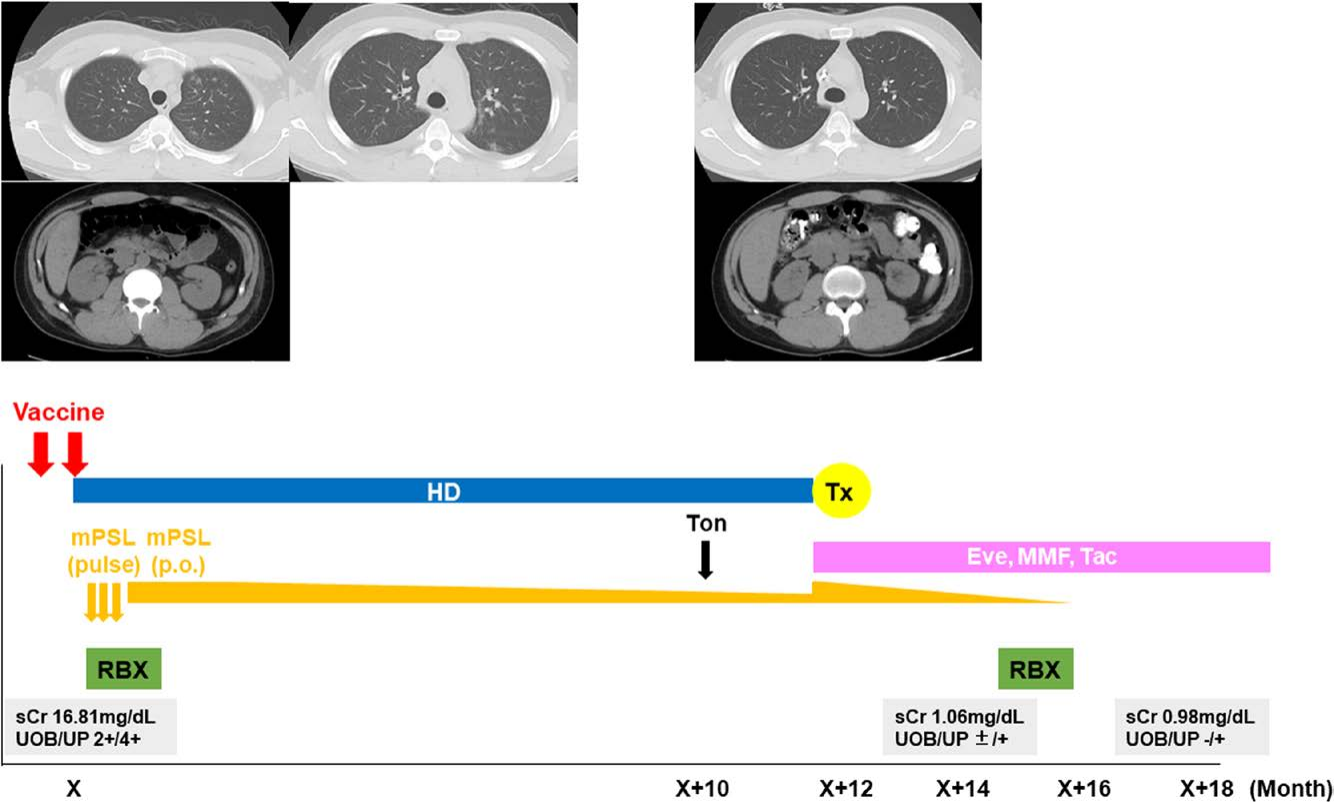
Patient’s clinical course. Computed tomography images taken on the first visit show ground-glass appearance in lung fields and enlarged kidneys (left panels). A computed tomography scan performed before renal transplantation reveals disappearance of the lung shadow (right panels). At this time, the kidneys were somewhat atrophic. Eve = everolimus; HD = hemodialysis; MMF = mycophenolate mofetil; mPSL = methylprednisolone; RBX = renal biopsy; sCr = serum creatinine; Tac = tacrolimus; Ton = tonsillectomy; Tx = transplant; UOB = urinary occult blood; UP = urinary protein.

The patient’s vital signs were as follows: body temperature, 37 °C; blood pressure, 184/124 mm Hg; and SpO_2_, 99%. Edema in both lower legs and conjunctival anemia were observed. The initial laboratory results are summarized in Table 1. Severe renal failure accompanied by dysmorphic hematuria and massive proteinuria were noted. Hypocomplementemia was absent, and autoantibodies, including anti-neutrophil cytoplasmic antibody and anti-glomerular basement membrane (GBM) antibody, were negative. Computed tomography showed ground-glass appearance in the lungs, suggesting alveolar hemorrhage, as well as enlarged kidneys (Fig. 1).

**Table 1:** Laboratory data of the patient at the first visit.

Urinalysis	Hematuria	5–9/HPF
	Proteinuria	5.22 g/day
*Complete blood count*	White blood cell (4000–8000/µL)	6940/µL
	Hemoglobin (13.5–17.5 g/dL)	7.7 g/dL
	Platelet (150,000–350,000/µL)	194,000/µL
*Biochemistry*	Creatinine (0.65–1.07 mg/dL)	16.81 mg/dL
	Blood urea nitrogen (8–20 mg/dL)	106.6 mg/dL
	Total protein (6.6–8.1 g/dL)	5.8 g/dL
	Albumin (4.1–5.1 g/dL)	2.6 g/dL
*Serology*	IgG (861–1747 mg/dL)	806 mg/dL
	IgA (93–393 mg/dL)	282 mg/dL
	IgM (33–183 mg/dL)	105 mg/dL
	Complement C3 (73–138 mg/dL)	93.2 mg/dL
	Complement C4 (11–31 mg/dL)	27.0 mg/dL
	Antinuclear antibody (<40)	<40
	MPO-ANCA (<3.5 U/mL)	0.0 U/mL
	PR3-ANCA (<3.5 U/mL)	0.0 U/mL
	Anti-GBM antibody (<3.0 U/mL)	<2.0 U/mL
	C-reactive protein (≦0.14 mg/dL)	1.14 mg/dL

ANCA = anti-neutrophil cytoplasmic antibody, GBM = glomerular basement membrane, HPF = high-power field, Ig = immunoglobulin, MPO = myeloperoxidase, PR3 = proteinase 3.

As the patient had presented with pulmonary–renal syndrome, intravenous methylprednisolone pulse therapy (500 mg/day for 3 days) was initiated from day 2 of hospitalization. Hemoptysis subsequently resolved whereas renal function worsened. Hemodialysis was initiated from day 5 of hospitalization, and oral methylprednisolone administration (40 mg daily) was continued.

**Figure 2.**
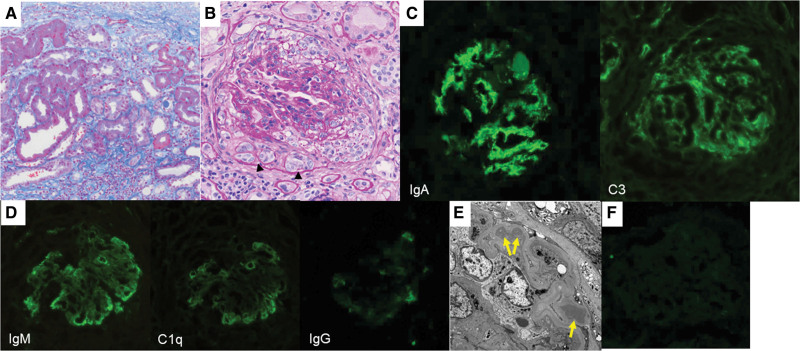
Histological features of the native kidney. (A) A high degree of interstitial fibrosis and tubular atrophy is seen (Masson trichrome stain). (B) A glomerulus showing cellular crescent and mesangial proliferation (periodic acid–Schiff stain). Pseudotubules formation is also observed (black arrowheads). (C) Immunofluorescence (IF) staining reveals deposition of immunoglobulin (Ig)A and complement C3 in the mesangial areas. (D) IgM and complement C1q deposition are seen as a peripheral pattern; deposition of IgG is negative. (E) Electron microscopy shows electron-dense deposits in the mesangial and paramesangial areas (yellow arrows), which correspond to IF depositions of IgA and C3. (F) Indirect IF staining to evaluate the presence of anti-glomerular basement membrane IgA-type antibody in the patient’s serum. A section of control renal tissue was incubated with the patient’s serum and then with fluorescein isothiocyanate-conjugated secondary antibody against human IgA (MBL, Tokyo, Japan), revealing negative results.

To determine the etiology of the pulmonary–renal syndrome, percutaneous renal biopsy was performed on day 8 of hospitalization. Light microscopy showed sections containing 37 glomeruli, among which 27 were obsolescent. Additionally, a high degree of interstitial fibrosis and tubular atrophy was present (Fig. 2A). Furthermore, all but one remaining glomerulus showed crescent formation accompanied by focal mesangial proliferation (Fig. 2B). Immunofluorescence staining revealed deposition of IgA and complement C3 in the mesangial area (Fig. 2C) and IgM and complement C1q as a peripheral pattern (Fig. 2D). Moreover, corresponding electron-dense deposits were found in the mesangial areas on electron microscopy (Fig. 2E). To rule out IgA-type anti-GBM disease, control tissue staining using the patient’s serum was performed as previously described.^[7]^ Briefly, a section of control renal tissue was incubated with the patient’s serum, washed with phosphate-buffered saline, and incubated with a fluorescein isothiocyanate-conjugated goat anti-human IgA antibody; a negative result was obtained (Fig. 2F). On the basis of these pathological findings and the absence of other typical manifestations of IgA vasculitis, a diagnosis of IgA nephropathy complicated by alveolar hemorrhage was made.

The clinical course of the patient is shown in Figure 1. The patient could not be weaned off dialysis therapy and was subsequently maintained on hemodialysis. Approximately 9 months after the patient’s first visit to our hospital, he was infected with SARS-CoV-2, but the infection was mild. Tonsillectomy was performed, and the patient underwent living-donor renal transplantation approximately 1 year after his first visit. Posttransplantation protocol biopsy, which was obtained about 3 months after the transplantation, showed almost normal renal tissue without evidence of rejection and recurrence of IgA nephropathy (Banff category 1; Fig. S1, Supplemental Digital Content, http://links.lww.com/MD/K680). The patient received monoclonal antibodies against SARS-CoV-2 (Evusheld [tixagevimab–cilgavimab]) to prevent COVID-19 infection. The patient is currently well; he has a normal serum creatinine level (approximately 1.0 mg/dL) and is maintained on everolimus, mycophenolate mofetil, and tacrolimus.

## 3. Discussion

To our knowledge, this is the first reported case of acute exacerbation of IgA nephropathy complicated by alveolar hemorrhage following COVID-19 vaccination. Although the patient did not have any significant medical history up to at least 5 years ago, the presence of advanced histological chronicity, which was demonstrated by renal biopsy tissue, suggested that he had undiagnosed and preexisting IgA nephropathy exacerbated by COVID-19 vaccination. IgA nephropathy-related alveolar hemorrhage is a rare condition that has high mortality and morbidity despite multifaceted approaches.^[6]^ Immunosuppression and/or plasmapheresis is often performed to treat pulmonary–renal syndrome; however, we did not perform either as the patient’s hemoptysis resolved following steroid monotherapy and there was a low likelihood of renal function recovery, as suggested by the renal biopsy findings. Immunofluorescence staining using the patient’s serum did not show linear IgA binding to the glomerular capillary walls (Fig. 2F), and the possibility of anti-GBM disease mediated by IgA antibodies was unlikely.^[7]^

There have been many reported cases showing the development or relapse of renal diseases following COVID-19 vaccination, with MCD and IgA nephropathy as representative disease. mRNA-1273 vaccines, which our patient received, reportedly increase the risk of developing or having a recurrence of IgA nephropathy, whereas BNT162b2 vaccines are associated with MCD.^[3,8]^ The widespread use of mRNA vaccines could affect the prevalence, but mRNA vaccines induce stronger immune responses than those induced by inactivated viral vaccines or natural infections.^[4]^ Additionally, mRNA COVID-19 vaccination reportedly induces early IgA antibody production^[9]^ and causes injury to glomerular endothelial cells.^[10]^

A single dose of a COVID-19 vaccine does not affect the risk for flare-ups of IgA nephropathy; however, there is a 3% increase in the risk for disease flare-ups following a second or third COVID-19 vaccination.^[11]^ Patients typically present with signs of exacerbation of IgA nephropathy within 1 or 2 days after receiving COVID-19 vaccination.^[4]^ Gross hematuria is the most common clinical presentation in these patients, followed by acute kidney injury.^[12]^ A survey in Japan reported that gross hematuria appeared within 3 days of vaccination in most cases.^[5]^ The benefits of COVID-19 vaccination is now considered to outweigh the risks, including the development or relapse of renal diseases, because most reported cases followed a mild or self-limiting clinical course.^[3–5]^ On the contrary, there have been reports of IgA nephropathy following COVID-19 vaccination that presents as acute exacerbation^[13–15]^ or rapidly progressive glomerulonephritis necessitating dialysis therapy.^[16]^ Further accumulation of cases of COVID-19 vaccination-related severe renal diseases is required to determine individuals at high risk. Monoclonal antibody against SARS-CoV-2 is effective in preventing COVID-19^[17]^ and is considered a potential option for patients who are difficult to receive COVID-19 vaccines, similar to our patient.

Tonsillectomy is widely performed in Asian countries, especially in Japan, as a fundamental treatment of IgA nephropathy in consideration of focal infection.^[18]^ Additionally, tonsillectomy is associated with a lower rate of recurrent IgA nephropathy after renal transplantation.^[19]^ However, whether tonsillectomy is effective for the treatment of IgA nephropathy complicated by alveolar hemorrhage or not remains unclear. It has been reported that recurrence with worsening renal function or recurrent alveolar hemorrhage occurs in more than one-third of patients with this rare disease even after initiating immunosuppressive therapy.^[6]^ Longer follow-up of the present patient to monitor for recurrence is therefore indispensable.

In conclusion, we have reported a case of IgA nephropathy complicated by alveolar hemorrhage following COVID-19 vaccination, suggesting that although extremely rare, severe renal failure requiring renal replacement therapy could occur after COVID-19 vaccination. Future studies to predict the risk of renal injury following vaccination are needed.

## Acknowledgments

We thank our colleague Ms. Sachiko Iwama for expert technical assistance in histological analyses, Ms. Yukari Kawamura, for expert secretarial assistance, and Editage (www.editage.jp), for English-editing of the manuscript.

## Author contributions

**Conceptualization:** Takahiro Uchida, Muneharu Yamada, Hitoshi Iwamoto, Takashi Oda.

**Data curation:** Takashi Sakai, Takahiko Hoshino, Aki Kojima, Osamu Konno, Hitoshi Iwamoto.

**Supervision:** Muneharu Yamada, Hitoshi Iwamoto, Takashi Oda.

**Writing – original draft:** Takahiro Uchida.

**Writing – review & editing:** Takashi Oda.

## Supplementary Material


