# Supplementary figures and images for: Acute exacerbation of immunoglobulin A nephropathy complicated by alveolar hemorrhage after coronavirus disease 2019 vaccination: A case report
Source: Medicine (Baltimore). 2023 Nov 17;102(46):e36091. doi: 10.1097/MD.0000000000036091 (PMC10659679; doi:10.1097/MD.0000000000036091)

## Slide 1
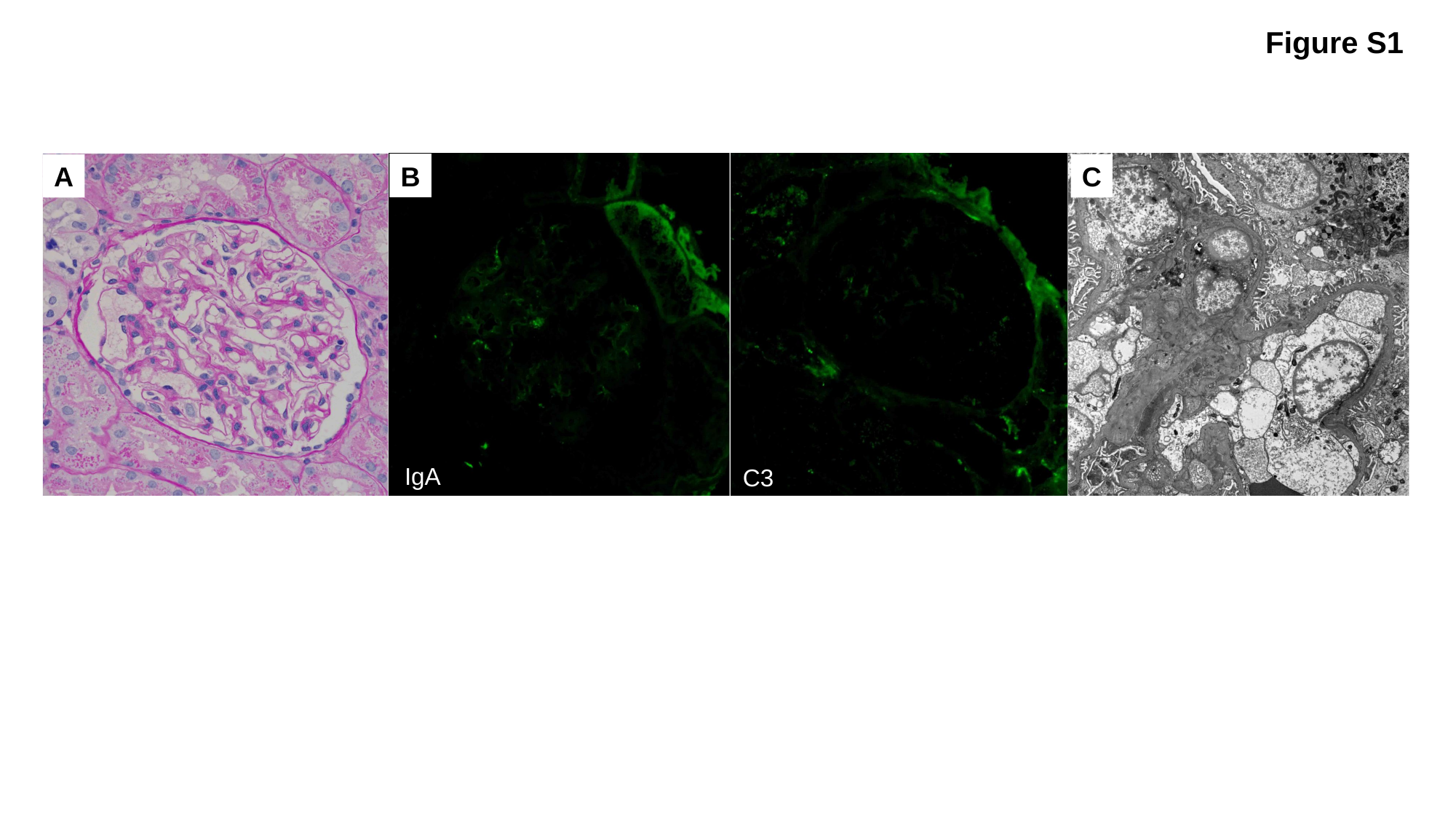

Figure S1
B
C
A
IgA
C3

Supplement: Supplementary file 1 [file medi-102-e36091-s001.pptx]
